# Metastability in lipid based particles exhibits temporally deterministic and controllable behavior

**DOI:** 10.1038/srep09481

**Published:** 2015-03-30

**Authors:** Guy Jacoby, Keren Cohen, Kobi Barkan, Yeshayahu Talmon, Dan Peer, Roy Beck

**Affiliations:** 1 The Raymond and Beverly Sackler School of Physics and Astronomy, Tel Aviv University, Tel Aviv 6997801, Israel; 2 Laboratory of NanoMedicine, Department of Cell Research and Immunology, George S. Wise Faculty of Life Sciences, Department of Materials Sciences and Engineering, Faculty of Engineering, Tel Aviv University, Tel Aviv 6997801, Israel; 3 Department of Chemical Engineering and the Russell Berrie Nanotechnology Institute (RBNI), Technion-Israel Institute of Technology, Haifa 3200003, Israel

## Abstract

The metastable-to-stable phase-transition is commonly observed in many fields of science, as an uncontrolled independent process, highly sensitive to microscopic fluctuations. In particular, self-assembled lipid suspensions exhibit phase-transitions, where the underlying driving mechanisms and dynamics are not well understood. Here we describe a study of the phase-transition dynamics of lipid-based particles, consisting of mixtures of dilauroylphosphatidylethanolamine (DLPE) and dilauroylphosphatidylglycerol (DLPG), exhibiting a metastable liquid crystalline-to-stable crystalline phase transition upon cooling from 60°C to 37°C. Surprisingly, unlike classically described metastable-to-stable phase transitions, the manner in which recrystallization is delayed by tens of hours is robust, predetermined and controllable. Our results show that the delay time can be manipulated by changing lipid stoichiometry, changing solvent salinity, adding an ionophore, or performing consecutive phase-transitions. Moreover, the delay time distribution indicates a deterministic nature. We suggest that the non-stochastic physical mechanism responsible for the delayed recrystallization involves several rate-affecting processes, resulting in a controllable, non-independent metastability. A qualitative model is proposed to describe the structural reorganization during the phase transition.

Classical nucleation and growth theories^1^, which describe the formation of a stable state preceded by a metastable state, are based in the framework of stochastic processes. These theories comprise microscopic details such as nucleation sites, boundary conditions, and density fluctuations, all of which are mediated by short-range interactions. In the absence of external perturbations, the metastable state energy-barrier can be overcome by spontaneous thermal fluctuations. Therefore, in bulk, nucleation results in an unpredictable and uncontrollable phase-transition.

The study of mesophases and polymorphism in lipids brought forth a wide variety of systems displaying rich phase-diagrams, including many examples of long-lived metastable states^2^,^3^. These systems feature rapid phase-transitions, as in the case of the gel-to-liquid-crystalline phase-transition, and gradual phase-transitions with slow dynamics. However, studying these dynamics can prove challenging, as they are often based on limited and qualitative observations.

One example of the metastable-to-stable phase-transition was reported for fully hydrated dispersions of dilauroylphosphatidylethanolamine (DLPE). The existence of a metastable liquid-crystalline (L_α_) to stable crystalline (L_c_) phase-transition upon cooling the dispersions below 43°C was reported, using DSC and x-ray scattering^4^,^5^,^6^. The L_α_ phase was suggested to be metastable until spontaneously reverting to the L_c_ phase upon incubation, in accordance with the stochastic aspect of classical nucleation theories.

The L_α_ and L_c_ phases are distinguished by their structure, degrees of freedom in motion, and lateral order. The L_c_ phase, stable at low temperatures, has a well-defined 3D structure. The high temperature L_α_ phase is characterized by lamellar correlations of the lipid bilayers and liquid disorder within them^7^. Membrane phase and correlation lengths can be analyzed using several techniques, including cryogenic transmission electron microscopy (cryo-TEM) and solution x-ray scattering (SXS). The latter directly measures the average correlation lengths, seen as scattering intensity peaks, corresponding to repetition abundances and long-range order.

Here, we experimentally investigate the L_α_ to L_c_ phase-transition dynamics of lipid-based particles (LPs) containing DLPE and dilauroylphosphatidylglycerol (DLPG). We find that these dynamics have a non-independent, cooperative, and controllable nature, therefore they do not conform to classical nucleation and growth theories.

## Results

**Figure 1:**
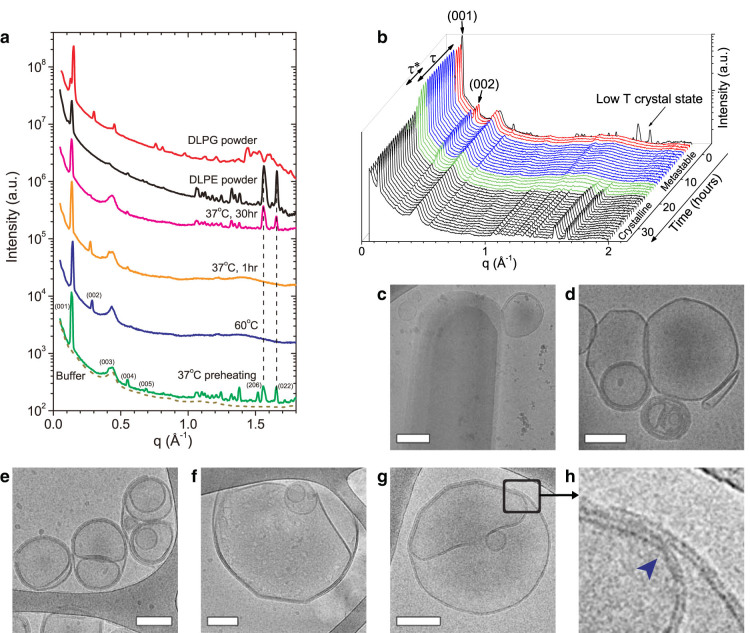
Time-resolved structural analysis of LPs showing delayed crystallization. (a) Representative SXS data at different stages of the experiment, compared with DLPE and DLPG powders. Crystalline peaks of the lipid aqueous dispersions match those of pure dry DLPE. (b) Initially, at 37°C (black spectrum), the crystalline state shows wide-angle peaks. When heated to 60°C (red spectra) the wide-angle peaks disappear with the loss of in-plane lipid order. After cooling back to 37°C (blue spectra) the L_α_ phase remains for 16.1 hours and then phase-transitions back into the crystalline state (green spectra). (c–h) Cryo-TEM images of specimens vitrified at different stages. (c) Specimen vitrified from 37°C prior to heat treatment, showing a large DLPE crystal structure along with uni- and multi-lamellar vesicles. (d) Sample vitrified from 37°C prior to heat treatment showing vesicles with sharp facets. (e) Sample vitrified from 60°C showing large MLVs. (f) Sample vitrified from 37°C 44 hours after cooling. Note the sharp facets forming on a vesicle. (g) Sample of a 90:10 DLPE:DLPG (mole %) vitrified from 37°C three days after cooling, showing multiple facets on a vesicle. (h) Detail of the previous micrograph showing the fusion of two membranes, accompanied by the removal of water from between them. Blue arrowhead indicates the fusion point of the two membranes, separating the crystalline phase (straight facets to the left) from the liquid-crystalline phase (disjoined, curved leaflets to the right). Unless specified, data are of 95:5 DLPE:DLPG (mole %) dispersions in 150 mM monovalent salt. Scale bar in (c) corresponds to 200 nm. Scale bars in (d–g) correspond to 100 nm.

Fully hydrated LP dispersions display a crystalline x-ray scattering pattern at 37°C. The scattering profiles are characterized by lamellar correlation peaks at small angles, and in-plane correlation peaks at wide angles (Fig. 1a, b). Cryo-TEM images of samples vitrified at 37°C revealed a population of large LPs and vesicles with straight facets (Fig. 1c, d). This is a distinct feature of the stable crystalline phase of DLPE, seen below the melting temperature (43°C). Upon heating to 60°C, the lipid hydrocarbon chains melt, and the membranes adopt the L_α_ phase, characterized by spherical, onion-like geometry (Fig. 1e). The liquid-crystalline phase brings about the loss of in-plane correlations, observed as the disappearance of the wide-angle peaks in the scattering profile (Fig. 1a).

When cooling back to 37°C the L_α_ phase remains, and becomes metastable. To study the metastability dynamics, we performed time-resolved SXS measurements, and found that the scattering profile remains unchanged for tens of hours (Fig. 1b). The metastability ends in a collective phase-transition back to L_c_, with identical in-plane correlation lengths to the initial L_c_ phase (Fig. 1a, b). The transition is accompanied by a drastic drop in the lamellar scattering intensity, indicating reorganization into LPs with fewer lamellae on average. The ultrastructure determined by cryo-TEM confirmed recrystallization of the hydrocarbon chains through the appearance of straight facets along the LP edges (Fig. 1f, g), accompanied by the fusion of adjacent bilayers (Fig. 1h).

A comparison of the SXS profile of anhydrous DLPE and DLPG to that of a hydrated sample containing 95:5 DLPE:DLPG (mole %), showed matching correlation patterns for the mixed sample and pure DLPE (Fig. 1a). The correlation peaks can be indexed to belong to an orthorhombic lattice with unit cell parameters *a* = 9.5 Å, *b* = 7.7 Å, and *c* = 45.6 Å, in close agreement with previous fully hydrated DLPE crystal indexation^4^,^5^ (see Supplementary Fig. S1).

The phase-transition dynamics show two distinct time scales: (a) the delay time, *τ*, which represents the metastability time span, and (b) the duration of the phase-transition, τ*, from when crystallization is first detected until completed (Fig. 1b). Both parameters can be quantified by fitting the x-ray correlation peak intensities using a sigmoid function (Supplementary Fig. S2). The step-like behavior of the metastability and the two separate time-scales imply that the dynamics are not governed by stochastic processes, as is expected from bulk nucleation phase-transitions. Furthermore, during the delay time, we did not observe any intermediate states, suggesting that the transition is directly between L_α_ and L_c_.

**Figure 2:**
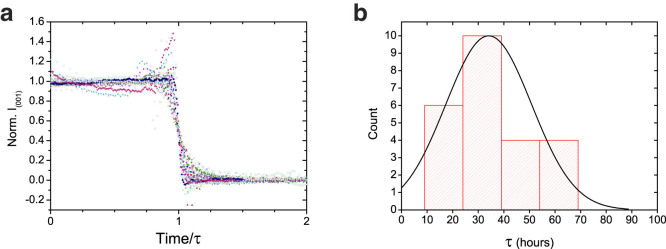
Reproducibility and universality of the delayed crystallization. (a) Intensity of the lamellar scattering peak as a function of time since cooling back down to 37°C. Intensity normalized to be between 1 (metastable state) and 0 (crystalline state after delayed transition), and time normalized by *τ*. A collapse of the entire data set, containing more than 60 experiments in varying conditions, highlights the robustness of the metastability dynamics and ensuing collective phase-transition. This emphasizes the governing role of *τ* in the dynamics. (b) Distribution of *τ* from measurements on 24 different samples. Black curve shows a Gaussian fit with an average delay time of <*τ*> = 34 hours and a standard deviation of 16.4 hours. The minimal recrystallization time (*τ*) measured was 9 hours. Experiments performed on samples containing 95:5 DLPE:DLPG at 150 mM monovalent salt.

Since the thermodynamic state of DLPE at 37°C is a dehydrated lamellar crystalline structure, perturbations applied initially (*e.g.*, sonication), a common method to treat lipid dispersions, would be absent at 37°C following the metastability. Thus, LPs at 37°C without additional homogenization or defect reducing procedures were chosen as the starting point for the investigation of the metastability. In fact, DLS measurements could not determine the size distribution of the LPs, indicating high polydispersivity in the samples. Supported by cryo-TEM images, we were able to detect that initial crystalline structures range from 0.5 to >10 μm in size. Nevertheless, the dozens of different samples prepared in this study showed the delayed metastability in a robust and reproducible manner (Fig. 2a). This suggests that the LP size-distribution is not a restricting condition for the appearance of delayed nucleation.

To address reproducibility and to test its nature, we show the delay time distribution of 24 samples containing 95:5 DLPE:DLPG (mole %) at near physiological conditions (Fig. 2b). The distribution has an average delay time of <*τ*> = 34 hours, and a standard deviation of 16.4 hours, resembling the average phase-transition time <τ*> = 15.4 hours.

If the metastability were to stem from a stochastic Poisson process, as expected from a classical barrier-hopping model^8^, the distribution of the time until crystallization would be exponential. However, the shape of the distribution is a broad Gaussian curve, and moreover, there were no occurrences of the delay time being shorter than 9 hours. We assume that partial insolubility of DLPE may lead to uncontrolled polydispersity in the desired lipid ratio, and could be the cause for a wide delay time distribution.

The observed delayed nucleation and the characteristic time-scales for crystallization raise important questions: What defines the time scales? Can the recrystallization delay time be manipulated? What is the physical origin of these unexpected metastability dynamics? We found that these dynamics can be manipulated by changing external parameters, such as lipid stoichiometry, solvent salinity, the presences of cross-membrane ion-carriers, and applying consecutive thermal cycles. The physical mechanisms responsible for the metastability are discussed later.

**Figure 3:**
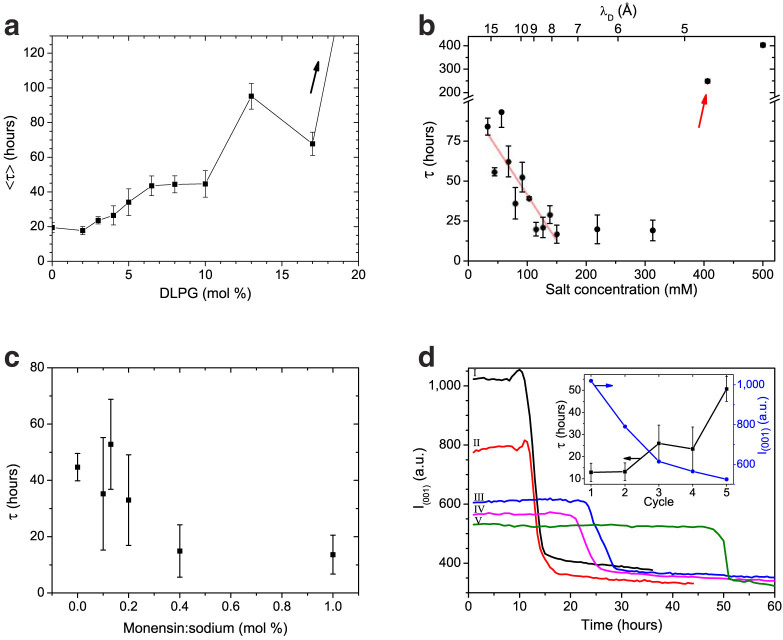
Manipulation of the delay time for crystallization at 37°C. (a) Average delay time increases with the addition of DLPG. Lipid dispersions in buffer containing 150 mM monovalent salt. The last point did not recrystallize in the duration of the experiment (200 hours). (b) Non-monotonic effect of salt concentration on the delay time, *τ*. With the addition of salt, at low concentrations (<150 mM), *τ* decreases in a linear fashion (red line), while at high concentrations (>300 mM), there is an order of magnitude increase. (c) Effect of the monensin ionophore on the delay time. Control sample recrystallized at *τ* = 44.7 hours. The addition of monensin accelerates recrystallization; sample containing 1 mole % monensin:sodium recrystallized with *τ* = 13.6 hours. Experiments performed on samples containing 90:10 DLPE:DLPG at 150 mM monovalent salt. (d) Consecutive heating-cooling cycles display a prolongation of the delay time accompanied by a decrease in the metastable lamellar scattering intensity. The curves represent the intensity of lamellar (001) scattering from the moment the temperature is brought back to 37°C after heating. Roman numbers indicate the measurement sequence. Inset shows qualitative analysis of the two effects. (b and d) represent data of 95:5 DLPE:DLPG (mole %). Error bars represent the average phase transition time <τ*>.

We included the anionic DLPG as a minor lipid component to assist in dispersing and stabilizing DLPE lipid structures, as reported by studies on drug delivery systems^9^,^10^,^11^. However, this addition had a striking effect on the metastability delay time (Fig. 3a). Increasing the amount of DLPG in the system prolonged the average *τ* from 19.5 hours, for pure DLPE, up to more than 200 hours at 20 mole % DLPG.

Although the lipid dispersions are charged (Supplementary Fig. S3), the out-of-plane distance between the lamellae is not stabilized by electrostatic repulsion (Supplementary Fig. S4). Instead, short-ranged van der Waals attractions between membranes are balanced by the hydration repulsion^12^, and long-range membrane undulations^13^. The former is pronounced for membranes separated by a distance of 30 Å or less, as is the case with the water layer thicknesses found between hydrated DLPE membranes^14^,^15^ (Supplementary Information). The latter is an entropic repulsion, typical of membranous systems at finite temperatures.

Nevertheless, monovalent salt (NaCl) concentrations affect the metastability non-monotonically (Fig. 3b). For example, in samples containing 95:5 DLPE:DLPG (mole %), at low salt concentrations (30–150 mM) an increase in the concentration resulted in a linear decrease in *τ*. Increasing the concentration furthermore (150–300 mM) had a minor effect. Yet, increasing the concentration even further (>300 mM) resulted in an increase by a factor of ten with *τ* = 402.8 and τ* = 15.4 hours for the sample containing 500 mM monovalent salt.

With salinity having a profound effect on *τ*, we incorporated monensin, a sodium specific ionophore, into the lipid dispersions. The control sample, containing 90:10 DLPE:DLPG (mole %) without monensin, recrystallized at *τ* = 44.7 hours and τ* = 9.7 hours. With the addition of monensin, we observed a monotonic decrease by up to a factor of three in the delay time (Fig. 3c).

Thermal cycles, above and below the melting transition temperatures, are commonly used to homogenize lipid samples. Apparently, such a treatment has a remarkable effect on the metastable state, and can be used to manipulate the crystallization delay time. We performed consecutive heating and cooling cycles to monitor successive phase-transition dynamics, using time-resolved SXS, and observed two distinct features. Successive cycles show a prolongation of the delay time, up to a factor of five in the 5^th^ cycle, and a reduction in the lamellar correlation peak intensity, measured at the metastable state (Fig. 3d).

## Discussion

Our results show that DLPE:DLPG dispersions exhibit a metastable state with a defined delay time prior to a cooperative phase-transition. This is in contrast to the stochastic framework of classical nucleation theories. The order-of-magnitude difference between the two time scales, *τ* and τ*, suggests different mechanisms for nucleation and growth, yet both are positively correlated (Supplementary Fig. S5). The phase-transition occurs on a short time scale compared to that of the delay time, implying a collective and cooperative crystallization event. To emphasize this behavior, we performed a global fit on all the time-resolved lamellar scattering profiles, constraining the exponent of the sigmoid (Supplementary Fig. S2). The fit produced an exponent of *n* = 48.5 ± 0.6, indicating a sharp transition. Moreover, rescaling the time by *τ*, and the intensity by the intensity in the initial metastable state, our entire data set, containing more than 60 experiments in varying conditions, collapses into a single sigmoidal curve (Fig. 2a). This further illustrates the cooperativeness of the phase-transition, and the robustness of the metastability to different sample conditions.

Phase reversibility and metastability have long been a key point in the study of lipids and their thermotropic behavior^2^. It has become clear that lipids display different phase-transition pathways, depending on their thermal history^3^,^4^,^5^,^6^,^16^. Moreover, it is also known that a number of intermediate phases (*e.g.* interdigitated, tilted, and rippled phases) may appear during phase transitions^17^. However, we did not observe such intermediate states during the metastable period of our samples.

Previous studies have shown the dependence of aqueous dispersions of phosphatidylethanolamines (PEs) on their thermal history^6^,^16^. Specifically, DLPE is known to have an endothermic L_c_ to L_α_ phase-transition at a temperature of about 43°C, in samples that were not pre-heated above this transition temperature^4^,^5^ (*i.e.* no thermal history). However, samples that were pre-heated above 43°C, then cooled and rescanned, displayed a smaller endothermic transition at 30°C, identified as the L_β_ to L_α_ gel-to-liquid-crystalline main transition. The L_c_ to L_α_ transition is characterized by a larger change in enthalpy attributed to the hydration of the headgroups as well as the melting of the hydrocarbon chains.

It was also shown that the L_α_ or L_β_ phases would become metastable upon cooling below 43°C, before returning to the stable L_c_ phase^4^. Upon cooling to 37°C, the transition is directly between L_α_ and L_c_. The metastability of the L_α_ phase was suggested to be the result of slow kinetics for dehydration of the headgroups, required for the recrystallization. The energetically unfavorable dehydration is compensated by an increasing van der Waals attraction and hydrogen-bond formation between headgroups^4^,^14^.

In light of our results, we propose that there are several rate-affecting processes acting simultaneously, influencing recrystallization, and setting the delay time. At 37°C, the dehydrated DLPE crystalline structure has an orthorhombic unit cell (Fig. 1a, Supplementary Fig. S1). However, during the incubation of the sample at 60°C, the L_α_ phase is adopted, disrupting homogeneity and lattice symmetry as a result of water and salt ion penetration in between the crystal lamellae. Furthermore, we notice that the second-harmonic lamellar correlation peak is absent only for the crystalline phase^4^,^5^ (Fig. 1a). This is likely due to the unique electron density profile of the DLPE crystal bilayer, which results in a form factor with an extinguished (002) reflection (Supplementary information). At 60°C the crystal lattice symmetry breaks, removing this unique geometry, which in turn causes the second harmonic to appear (Fig. 1a). Maintaining a similar lamellar repeating distance, while removing water from between the lamellae during the phase-transition back to L_c_, is attributed to the elongation of the hydrocarbon chains^4^,^18^. We were able to demonstrate the above analytically, in which the inter-lamellar distance was kept constant, while the (002) reflection was reduced by orders magnitude upon removing the water (Supplementary Information). The correlation between the disappearance of the (002) out-of-plane reflection and the appearance of in-plane wide-angle reflections demonstrate that water removal between the layers and hydrocarbon crystallization are strongly linked.

Water molecules and salt ions delay crystallization via hydration repulsion and electrostatic interactions. They prevent the lipid bilayers from adhering and reforming the crystal lamellar structure when the temperature is lowered back to 37°C. By adding the monensin ionophore we increase the exit rate of ions from within the lamellae, which in turn accelerates recrystallization (Fig. 3c). The presence of water in itself is also widely considered a delaying factor due to slow kinetics^2^,^3^,^4^.

Another process that could hinder recrystallization is the elastic deformation needed to transform the spherical LPs to faceted crystalline structures at 37°C. The cryo-TEM images show the morphological evolution of the membranous structures, from a spherical geometry seen at 60°C (Fig. 1e), to growing facets on round vesicles (Fig. 1f, g), and finally to polygonal crystalline structures. A difference in spontaneous curvatures of DLPE and DLPG can introduce additional complexities for lateral mobility and curvature generation.

Yet another delaying process, also occurring during the liquification of the crystals, is the incorporation of DLPG into the DLPE bilayers. To restore the initial homogeneous state of the DLPE, the two lipids must segregate. However, DLPG is negatively charged and thus resists this process, preserving the L_α_ phase^19^. We attribute the increase of *τ* at high DLPG content and at low salt concentrations (*i.e.*, reduction of screening) to this segregation process (Fig. 3a, b). Moreover, the concurring lateral phase separation of the L_c_ and L_α_ phases, leads to long-range interlayer interactions^20^. This can serve an important role in the following comparison.

Delayed nucleation was observed and studied theoretically in the paraelectric to ferroelectric phase-transition in BaTiO_3_^21^,^22^,^23^. There, mediated by long-range interactions, the metastable activation energy-barrier was modeled as time-dependent, which ultimately defined the delay time of the system. The delay time was measured to be six orders of magnitude larger than the expected time-scale^21^,^23^. Similar to this example, the LPs' delay times are of orders of magnitude larger than the expected time-scales determined by stochastic processes such as lipid diffusion. Moreover, it is clear that upon cooling the LPs to 37°C the activation energy barrier for crystallization is too high to occur spontaneously. At low temperatures several simultaneously acting processes, described above, delay recrystallization for an extended period of time. We suggest that a slow transition to a 2D crystalline state^24^ in the lamellae lowers the activation barrier until 3D crystallization propagates throughout the entire sample.

**Figure 4:**
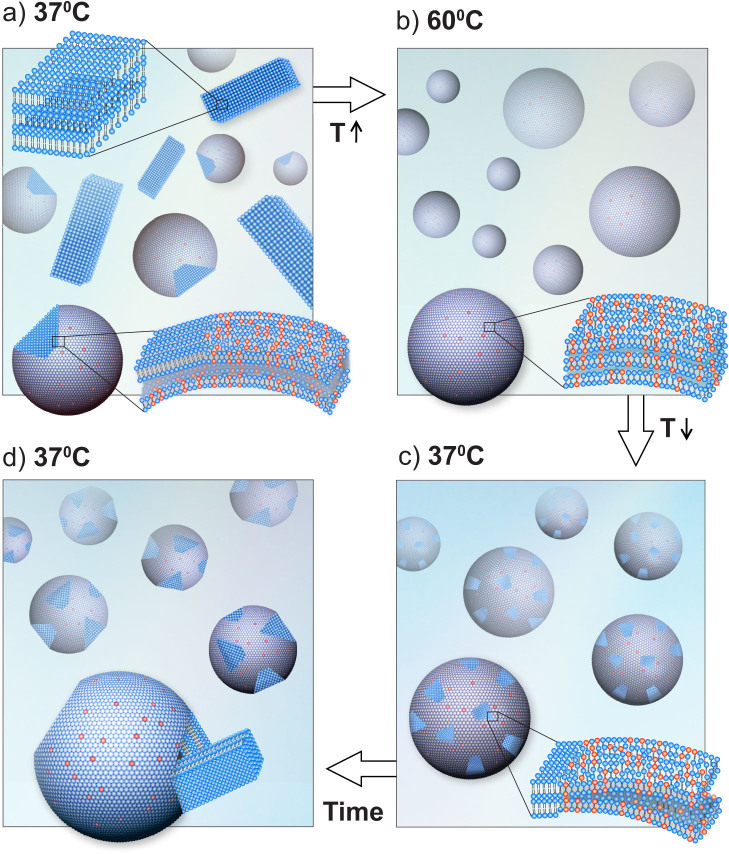
Schematic representation of the morphology during the different stages of the experiment. (a) At 37°C prior to heating, the system is composed of a population of large crystals and vesicles with facets. (b) After heating to 60°C, the lamellae adopt the L_α_ phase with water and ions penetrating between the membranes. (c) Cooling the system to 37°C, after heating, the membranes remain in the L_α_ phase and 2D to 3D facets begin to form and grow. (d) The crystallization phase transition occurs after large enough facets disrupt the morphology of the LPs. The structural reorganization and coexistence of DLPE crystals and mixed DLPE/DLPG LPs result in MLVs with fewer lamellae on average.

The morphological changes we observed reflect the nucleation and growth processes during the waiting period (*τ*). Recrystallization of DLPE on vesicles is seen in the cryo-TEM images as 2D facets. We suggest that the final stage of the forming 2D facets is expressed in a critical size of the shape reformation; the sharp facets increase in size, until the membrane can no longer retain its integrity (Fig. 4). Rupture of the membrane releases DLPE into solution, resulting in a reorganization of LPs into smaller structures containing fewer lamellae, seen as the drastic decrease in lamellar scattering intensity during the phase-transition (Fig. 1b).

In this study we have shown that lipid particles comprised of DLPE and DLPG exhibit a controllable delay time to crystallization. The deterministic behavior of the dynamics conflicts with the classical picture of stochastic nucleation and growth. Moreover, we found alternative routes to manipulate and control the delay time, such as lipid stoichiometry, monovalent salt concentration, ionophore inclusion, and consecutive thermal treatments. These findings open new avenues for research in metastability and controlled delayed nucleation using LPs. Finally, since DLPE:DLPG dispersions are currently studied as components of drug delivery nano-carriers^9^,^10^,^11^, manipulation of delayed nucleation may ultimately aid in designing novel modalities for entrapment of therapeutic payloads and their controlled release.

## Methods

### Lipid dispersion preparation

1,2-dilauroyl-sn-glycero-3-phosphoethanolamine (DLPE) and 1,2-dilauroyl-sn-glycero-3-glycerol (DLPG) were purchased from Avanti Polar Lipids Inc. The lipids were dissolved in chloroform (DLPE) and chloroform/methanol 5:1 (DLPG) separately, then mixed together to achieve desired stoichiometry. Total lipid concentration was 30 mg/ml per sample. The solution was evaporated overnight in a fume hood, and re-fluidized using a buffer at 6.7 pH containing 20 mM MES, 1 mM MgCl_2_ and 13 mM NaOH. NaCl was added to retain desired monovalent salt concentration. Samples were then placed in a 37°C bath for 3 hours, and homogenized using a vortexer every 25 minutes. Samples were then placed in quartz capillaries, containing about 40 μl, and centrifuged for 5 minutes at 3000 rpm, to create a pellet of lipids.

### Solution x-ray scattering experiments

Samples at 30 mg/ml lipid concentration were measured in 1.5 mm diameter sealed quartz capillaries. Measurements were performed using an in-house Solution X-Ray Scattering system, with a GeniX (Xenocs) low divergence Cu K_α_ radiation source (wave length of 1.54 Å) and a scatterless slits setup^25^. The samples were placed in a capillary temperature chamber (Forvis Technologies), with a temperature accuracy of ±0.1°C. Measurements were performed in 1 hour intervals, with a data collection time of 10 minutes each. The samples were first measured at 37°C, after equilibration, over a period of 2 hours. The samples were then measured at 60°C, after equilibration, over a period of 3 hours. The temperature was then brought back to 37°C, at a temperature change rate of about 6.5°C/minute, and immediately measured in sequence. Two-dimensional scattering data, with a *q* range of 0.06–2 Å^−1^ at a sample-to-detector distance of about 230 mm, was collected on a Pilatus 300 K detector (Dectris), and radially integrated using Matlab (MathWorks) based procedures (SAXSi). Background scattering data was collected from buffer solution alone. The background-subtracted scattering correlation peaks were fitted using a Gaussian with a linearly sloped baseline. For each sample, time-resolved correlation peaks position, intensity and width where extracted. Additional structural characterization was conducted, as described in the supplementary information.

### Particle size-distribution and zeta potential measurements

Lipids were dissolved as described in the lipid preparation section and mixed in the selected DLPE:DLPG (mole %) ratios: 100:0, 95:5, 90:10, and 85:15, to a final lipid concentration of 10 mg/ml. The organic solvents were evaporated in a fume hood overnight. The day after, the dry lipid film was reconstituted with MES buffer pH 6.7 (150 mM monovalent salts). The samples were heated to 37°C for 3 hours and homogenized using vortex every 25 minutes. Afterwards, samples were heated to 60°C for 1 hour. Samples were collected before and after heating to 60°C, diluted 1:50 in double distilled water and their hydrodynamic size and surface charge (zeta potential) were characterized using the ZetaSizer Nano ZS (Malvern Instruments Inc., UK), utilizing dynamic light scattering (DLS) and electrophoretic light scattering (ELS), respectively^26^,^27^. The instrument was pre-heated to 37°C. Three measurements were performed per each sample.

### Cryo-TEM imaging

Cryogenic transmission electron microscopy (cryo-TEM) specimens were prepared in a controlled environment vitrification system (CEVS), to preserve the native structure of the system at the desired temperature^28^. A drop of the solution was placed on a carbon-coated perforated polymer film, supported on a 200 mesh TEM copper grid, mounted on a tweezers. Thin liquid films (preferably less than 300 nm thick) were formed by blotting excess solution with a metal strip wrapped with a filter paper. The specimen was then plunged into liquid ethane at its freezing point (−183°C). We performed cryo-TEM imaging with an FEI Tecnai T12 G^2^ electron microscope, operated at an accelerating voltage of 120 kV. We transferred the cryo-specimens under controlled conditions into a Gatan 626DH cryo-holder, using its “transfer station”. After the specimens were equilibrated in the TEM below −175°C, we imaged them in the low-dose imaging mode to minimize electron-beam radiation-damage. We recorded the images digitally by a Gatan US1000 high-resolution cooled CCD camera, using the Gatan DigitalMicrograph software.

## Author Contributions

G.J., D.P. and R.B. designed the experiments. G.J. and K.C. prepared samples. G.J. and R.B. performed scattering experiments. G.J., K.B. and R.B. analyzed the scattering data. Y.T. performed cryo-TEM experiments. All authors discussed the results and wrote the manuscript.

## Supplementary Material

Supplementary Information Supplementary Information
