# Supplementary material for: Metastability in lipid based particles exhibits temporally deterministic and controllable behavior
Source: Sci Rep. 2015 Mar 30;5:9481. doi: 10.1038/srep09481 (PMC4377625; doi:10.1038/srep09481)
Supplement: Supplementary Information [file srep09481-s1.pdf]

<u>Metastability in lipid based particles exhibits temporally deterministic and controllable behavior - Supplementary Information</u>

Guy Jacoby, Keren Cohen, Kobi Barkan, Yeshayahu Talmon, Dan Peer, Roy Beck

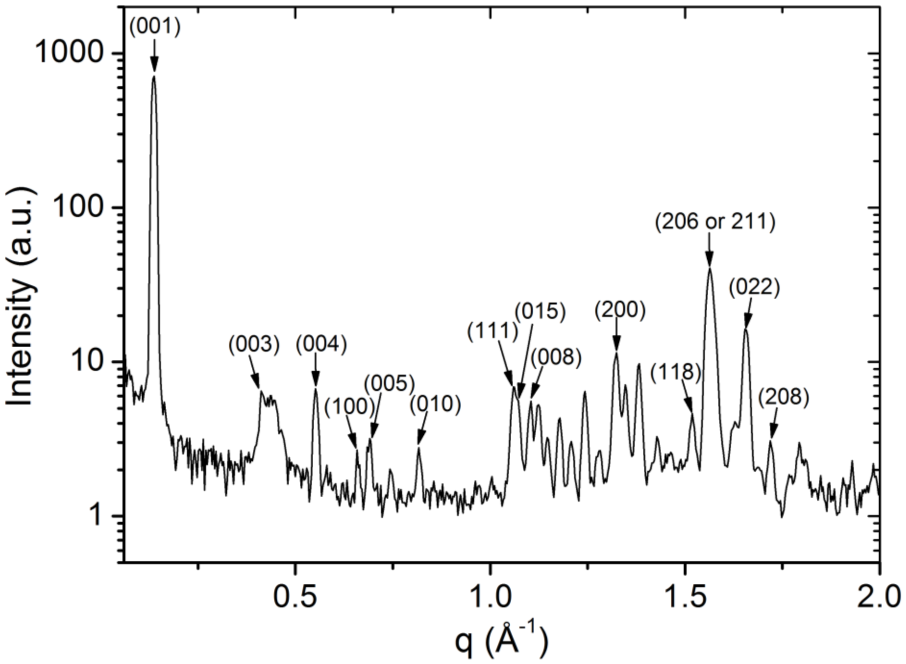

Supplementary Figure S1 | Crystal structure indexation. Integrated SXS of fully hydrated DLPE at 37 °C prior to heating. Correlation peaks indexed as an orthorhombic lattice with parameters a = 9.5 Å, b = 7.7 Å and c = 45.6 Å.

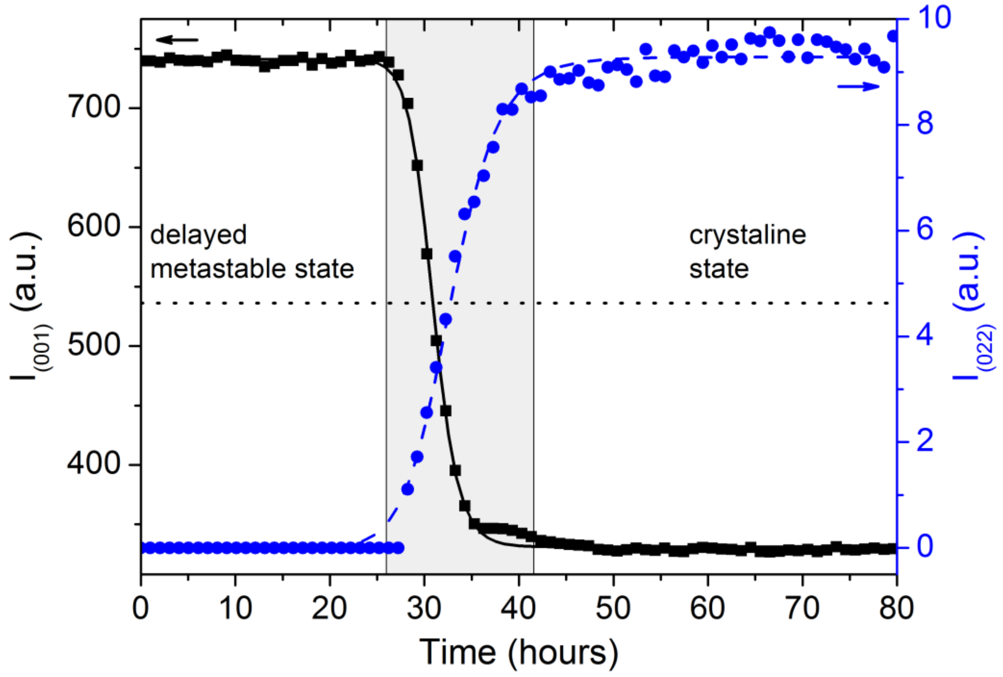

Supplementary Figure S2 | Example of fitted sigmoid function (lines) to peak intensities $I_{(001)}$ and $I_{(022)}$ as a function of time since cooling back to 37 °C. The fitted sigmoid function is $I(t) = I_0 + I_1\left(\frac{t^n}{t^n + \tau^n}\right)$, where I_0 is the metastable phase intensity, I_1 is the difference in intensity between the crystal state and the metastable phase, t is the elapsed time since the sample was cooled to 37 °C. Analysis and fitting are done after background subtraction and τ^* is extracted from the first derivative of the fitted results as the width at 1% of maximum.

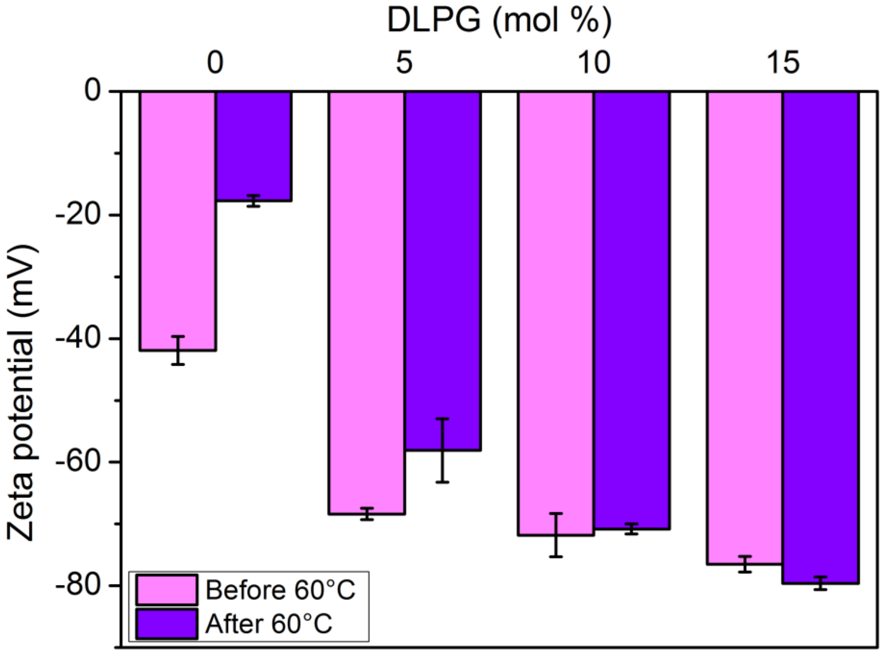

Supplementary Figure S3 | Zeta-potential measurements of mixed sample before and after heating to 60 °C. Increasing the charged lipid concentration results in a higher zeta-potential of the lipid particles. Three measurements were performed for each sample. Error bars represent the standard deviation of the measurements.

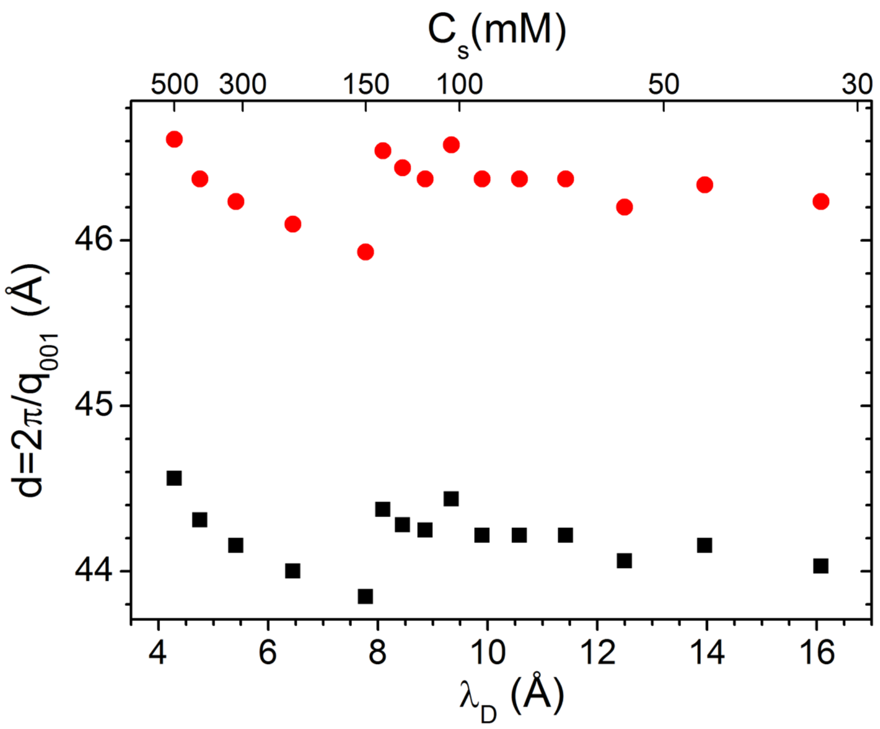

Supplementary Figure S4 | Inter-lamellar spacing vs. the Debye screening length or salt concentration. Increasing salt concentration did not have a noticeable effect on inter-lamellar spacing, both at 37 °C (red circles) and at 60 °C (black squares).

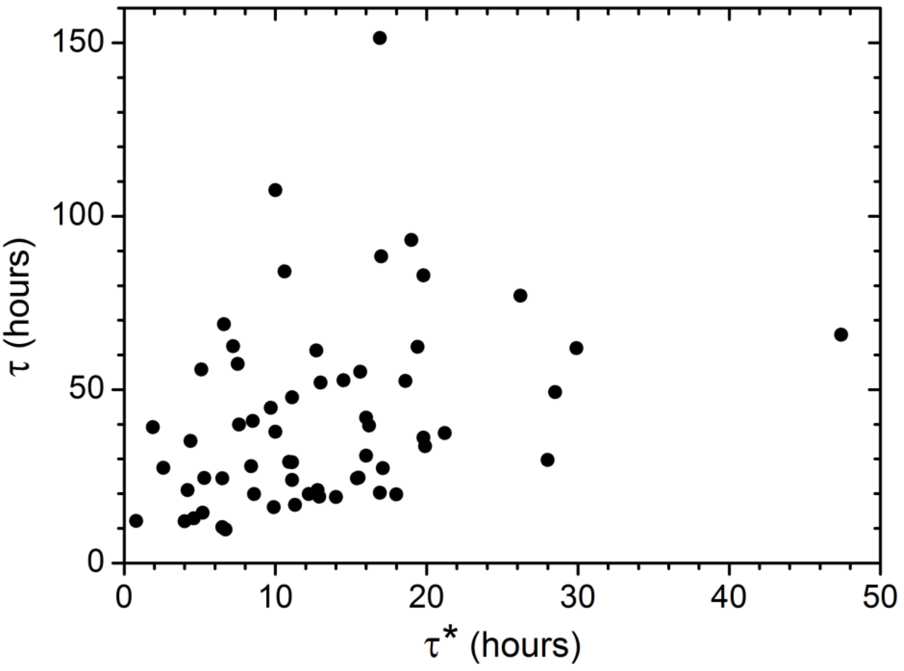

Supplementary Figure S5 | Delay time (τ) vs. transition time (τ^*). A positive correlation between the two time scales is observed.

Bilayer structural information.

Reconstructing the electron density profile of the lipid bilayer is an essential part in the study of uni- or multi-lamellar lipid systems. This can be achieved by means of a discrete Fourier synthesis, while sampling the continuous form factor at q-values pertaining to lamellar correlation peaks. However, reconstruction is also possible using a continuous method, which takes advantage of the full q-range scattering line shape. We applied such a continuous fitting method, which utilizes a modified Caillé theory structure factor in combination with a Gaussian model representing the electron density profile[1,2]. In this approach the diffuse scattering between Bragg peaks is taken into account, in addition to a divided contribution by multi- and uni-lamellar vesicles. Moreover, this method allows for the derivation of additional structural parameters.

In Supplementary Fig. S6 we show the result of the line-shape analysis and fitting to pure DLPE in solution, in the metastable L_α phase, a few hours after cooling from 60 °C to 37 °C. The fitting procedure produces an electron density profile (Supplementary Fig. S7) and fitted parameters (Supplementary Table 1). In addition, the derived structural parameters, which are in agreement with previous studies[3,4], are summarized in Supplementary Table 2.

The fitting process involves setting constraints for the fitting parameters. Those constraining values reflect physical/chemical knowledge of the system. Some of the parameters prove difficult to accurately assess for the convergence of the fit, such as the average number of lamellae per vesicle and the fraction of uni-lamellar vesicles (not contributing to the lamellar Bragg peaks). This degeneracy is probably due to the high polydispersity in the samples.

We noticed the scattering profile of mixed DLPE/DLPG dispersions show a widening of the Bragg peak bases with respect to the DLPG content (Supplementary Fig. S8). Such change in the scattering line-shape trend can be achieved by changing either the fluctuation parameter (η) or the Gaussian width representing the electron density of the hydrocarbon tails (σ_C). However, these lipids mixtures present a more complex system than the single component model used for the fit. Therefore the fitting procedure is unable to accurately model the physical changes occurring in the system with the addition of DLPG.

As mentioned in the text, the Bragg peak of the second harmonic, denoted (002), is absent only for the initial crystal phase[5,6] (Fig. 1a). In order to explain the disappearance of the (002) reflection in the crystallized state, we assume a unit cell of two identical lipid molecules comprising a symmetric bilayer of length d, with the molecules' chains facing each other in opposite directions. The form factors of both molecules are given by

(1) $f_1(k) = \int_0^{d/2} dz\, \rho(z) e^{-ikz}$,

(2) $f_2(k) = \int_{d/2}^{d} dz\, \rho(d-z) e^{-ikz} = e^{-ikd} f_1(-k)$.

Due to the electron density being real, it follows that

(3) $f_1(-k) = f_1^*(k)$.

Therefore, the intensity of the Bragg peak at $k = \frac{2\pi}{d} n$ is proportional to

(4) $\left(f_1(k) + f_1^*(k)\right)^2$.

Finally, the intensity of the second harmonic is given by

(5) $I_{002} = \left(\int_0^{d} dz\, \rho(z) \cos(2 * \frac{2\pi}{d} z)\right)^2$.

The removal of water from between the lamellae in the crystal state, while maintaining a similar repeating distance, can be described as the elongation of the hydrocarbon chains in an extended conformation of the lipid molecules. To account for the conformational change, we increased the width and lowered the amplitude of the hydrocarbon chains Gaussian, and increased the head-to-head distance. We find that by varying the head-to-head distance by less than 10%, while maintaining the area of the hydrocarbon Gaussian (within 1%), we are able to reduce the (002) reflection scattering profile by orders of magnitude. The altered fitting parameters for the crystal state are shown in Supplementary Table 1.

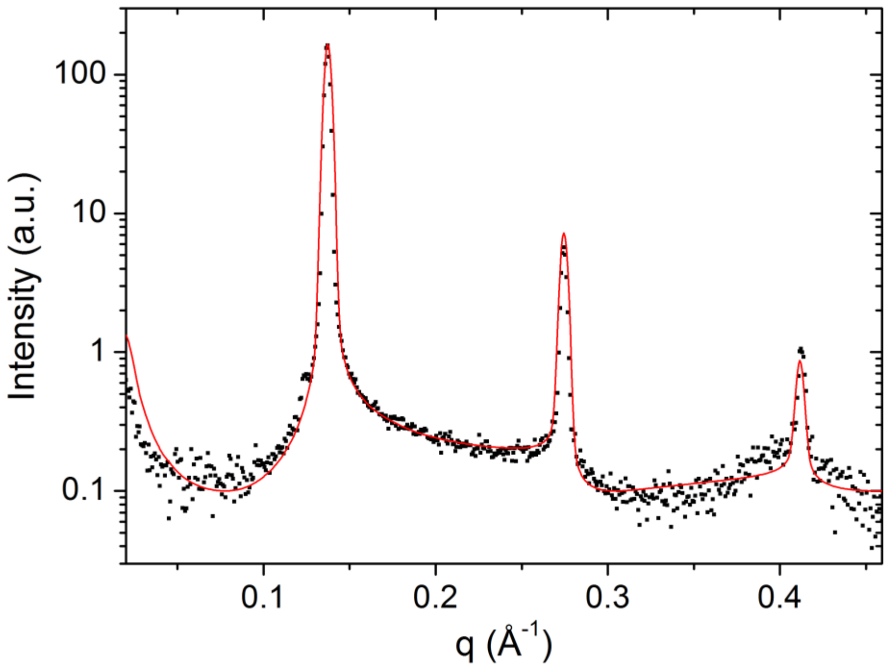

Supplementary Figure S6 | Full q-range fit using line-shape analysis. X-ray scattering data (points) and the line-shape analysis fit (red curve). Features like the diffuse scattering around Bragg peaks are taken into account in the fitting process. Discrepancies between the data and the fit arise due to the model's limited ability to account for a multi-component system with high polydispersity. Data of sample containing DLPE in 150 mM monovalent salt solution.

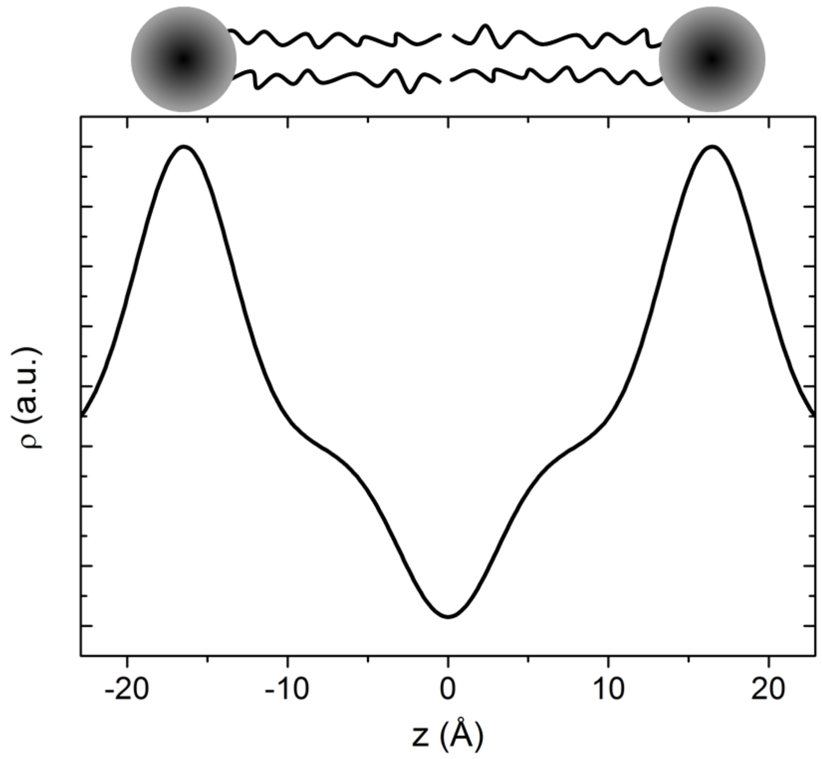

Supplementary Figure S7 | Bilayer electron density profile of DLPE. Profile generated from the fitted scattering curve using line-shape analysis (Supplementary Information). Sample contained 150 mM monovalent salt.

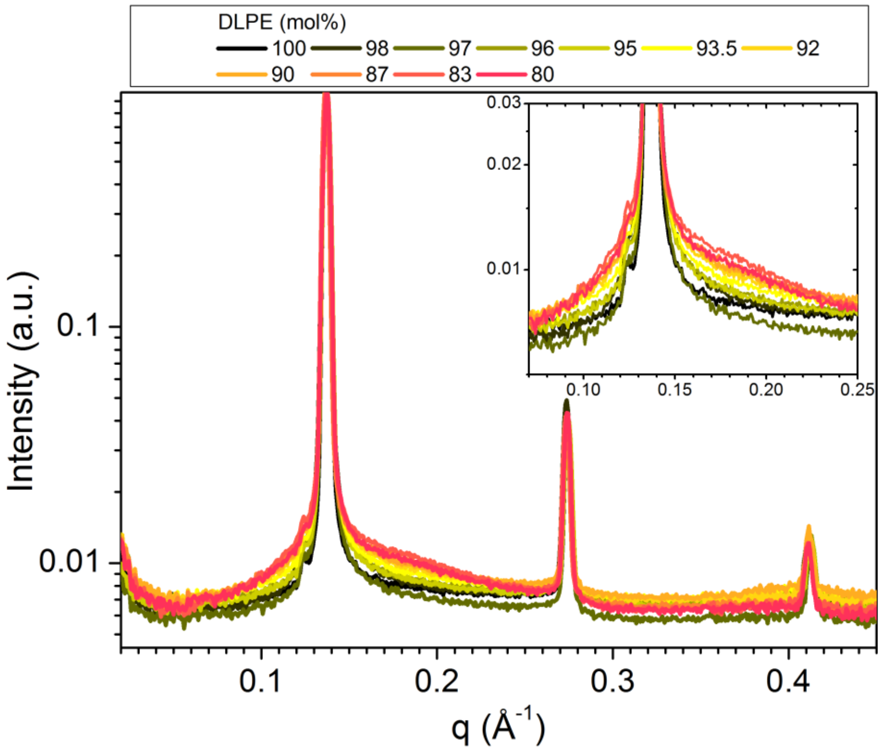

Supplementary Figure S8 | Scattering of mixed DLPE:DLPG samples. Widening of the Bragg peak bases with respect to the DLPG content is observed.

Fit parameter	T = 37°C	
	L_c	L_α
Z_H (Å)	17.8	16.46
σ_H (Å)	3	3
ρ_C (e/Å^3)	-0.354	-0.57
σ_C (Å)	5	3.064
d (Å)	45.78	45.78
η_1	0.01	0.017
N	297.4	297.4
N_{diff}	0	0

Supplementary Table 1. Fitting parameters for line-shape analysis both at the crystalline and metastable phases. Fitting parameters are presented as in refs. 1, 2. Right column (denoted L_α) displays the fitting parameter results for a sample at 37 °C 1 hour after cooling back from 60 °C. Left column (denoted L_c) displays the fitting parameters for a sample at 37 °C prior to heating, that diminish the (002) reflection as explained in the supplementary information text. Fitting performed on scattering data from samples containing 95:5 DLPE:DLPG (mol:mol%) in 150 mM monovalent salt solution, using GAP software provided by Dr. Georg Pabst and ref. 1 and 2.

Structural parameter	37°C		35°C[§]
	L_c	L_α	
Area per lipid (A)	45.4 Å^2	50.4 Å^2	51.2 Å^2
Bilayer thickness (d_B)	42.7 Å	40 Å	42.8 Å
Chain length (d_C)	14.3 Å	12.9 Å	12.9 Å
Water layer thickness (d_W)	3.1 Å	5.8 Å	5 Å
Total number of water molecules per lipid[†] (n_w)	7.7	10.8	8.8
Number of free water molecules per lipid[‡] (n^*_w)	2.3	4.9	4.1

[†]including water molecules inside the headgroup
[‡]water layer between opposing headgroups
[§]values taken from ref. 4. Steric parameters: D_B', D_C, D_W', (n_W - n_W')

Supplementary Table 2. Structural parameters derived from the fitting parameters in Supplementary Table 1, according to the procedures in ref. 1, along with the values given in ref. 4 for comparison.

References

1. Pabst, G., Rappolt, M., Amenitsch, H. & Laggner, P. Structural information from multilamellar liposomes at full hydration: Full q-range fitting with high quality x-ray data. *Phys. Rev. E* **62**, 4000–4009 (2000).

2. Pabst, G. et al. Structural analysis of weakly ordered membrane stacks. *J. Appl. Crystallogr.* **36**, 1378–1388 (2003).

3. McIntosh, T. J. & Simon, S. A. Area per molecule and distribution of water in fully hydrated dilauroylphosphatidylethanolamine bilayers. *Biochemistry* **25**, 4948–4952 (1986).

4. Nagle, J. F. & Tristram-Nagle, S. Structure of lipid bilayers. *Biochim. Biophys. Acta - Rev. Biomembr.* **1469**, 159–195 (2000).

5. Seddon, J. M., Harlos, K. & Marsh, D. Metastability and polymorphism in the gel and fluid bilayer phases of dilauroylphosphatidylethanolamine. Two crystalline forms in excess water. *J. Biol. Chem.* **258,** 3850–4 (1983).

6. Chang, H. & Epand, R. M. The existence of a highly ordered phase in fully hydrated dilauroylphosphatidylethanolamine. *Biochim. Biophys. Acta - Biomembr.* **728,** 319–324 (1983).
